# Dental and Surgical Instruments

**Published:** 1842-03

**Authors:** 


*Dental and Surgical Instruments.*—An opinion is entertained by some, that the manufacture of surgical and dental instruments has been brought to much greater perfection in Europe than in America, but this, we believe, is not well founded. There is no description of surgical operations, which, in their performance, require more finely tempered or highly finished instruments, than those that pertain to the teeth, and we have had frequent opportunities of comparing the instruments employed for the performance of these, manufactured at some of the first cutlery establishments in Europe with those manufactured in this country, and for excellence, both of temper and finish, we have no hesitation in pronouncing the preference to be due to the latter. We have cutlers in Philadelphia, New York, Baltimore and Boston, whose skill in the fabrication of surgical and dental instruments, we believe, to be unsurpassed. For the procurement of these, therefore, there is now no necessity for sending abroad. We had a pair of lower molar forceps presented to us a few days since, and there is no dental instrument, in the manufacture of which cutlers have more frequently failed to produce such as were really good, than this, made by Messrs. Daily & Arnold of Baltimore, that surpasses any thing of the kind we have ever before seen.

[*Balt. Ed.*

